# Architecture and mechanism of metazoan retromer:SNX3 tubular coat assembly

**DOI:** 10.1126/sciadv.abf8598

**Published:** 2021-03-24

**Authors:** Natalya Leneva, Oleksiy Kovtun, Dustin R. Morado, John A. G. Briggs, David J. Owen

**Affiliations:** 1 Cambridge Institute for Medical Research, Cambridge Biomedical Campus, Hills Road, Cambridge CB2 0XY, UK.; 2 MRC Laboratory of Molecular Biology, Cambridge Biomedical Campus, Cambridge CB2 0QH, UK.

## Abstract

Retromer is a master regulator of cargo retrieval from endosomes, which is critical for many cellular processes including signaling, immunity, neuroprotection, and virus infection. The retromer core (VPS26/VPS29/VPS35) is present on cargo-transporting, tubular carriers along with a range of sorting nexins. Here, we elucidate the structural basis of membrane tubulation and coupled cargo recognition by metazoan and fungal retromer coats assembled with the non–Bin1/Amphiphysin/Rvs (BAR) sorting nexin SNX3 using cryo–electron tomography. The retromer core retains its arched, scaffolding structure but changes its mode of membrane recruitment when assembled with different SNX adaptors, allowing cargo recognition at subunit interfaces. Thus, membrane bending and cargo incorporation can be modulated to allow retromer to traffic cargoes along different cellular transport routes.

## Teaser

Cryo–electron tomography shows how retromer recruits cargo and tubulates endosomes without canonical membrane-bending proteins.

## INTRODUCTION

The protein coat complex known as retromer is a central component of the endosomal sorting machinery (*1*, *2*). Endosomal sorting is key to cellular homeostasis, and its malfunction is associated with pathophysiological conditions including neurodegenerative disorders, with genetic causes being mapped to the retromer complex and its auxiliary proteins (*3*). Genome-wide screens have identified retromer as one of the most important host factors for SARS-CoV-2 (severe acute respiratory syndrome coronavirus 2) infection (*4*, *5*). The core of retromer is a trimer comprising vacuolar protein sorting (VPS) proteins VPS35, VPS26, and VPS29. Cryo–electron tomography revealed the architecture of fungal retromer core assembled on membranes via a membrane adaptor, a dimer of sorting nexins (SNX) containing Bin1/Amphiphysin/Rvs (BAR) domains, in which the core trimer forms an arch-like structure over a layer of SNX-BAR adaptors (*6*). BAR domains are known for their curvature-generating/stabilizing properties (*7*), and the accepted dogma is that membrane curvature enabling the formation of tubules is induced by the SNX-BARs, with retromer core trimer playing an auxiliary role (*8*).

The core trimer has been reported to assemble with other members of the SNX family of adaptors (*9*, *10*) to expand the repertoire of cargoes that can be sorted into retromer-coated tubules and to mediate additional trafficking routes that are distinct from the retromer:SNX-BARs pathway (*11*–*13*). In metazoa, other adaptors include SNX3, SNX12 (*14*, *15*), and SNX27 (*16*–*18*) that belong to different classes of proteins within the same SNX family. In fungi, the only known non–BAR-containing SNX adaptor is the SNX3 homolog, Grd19 (*13*, *19*). The complexity of retromer biology in metazoa likely reflects both the increased number of cargoes and the increase in diversity of trafficking routes from endosomes (*1*, *18*, *20*).

The current dogma contains a contradiction in that curvature generation is ascribed to the SNX-BAR adaptor proteins, but retromer’s ability to transport a wide range of cargoes has been ascribed to the use of a range of different adaptors, including those that do not contain membrane-bending BAR domains. This has led to the suggestion that metazoan and fungal retromer may adopt different architectures (*21*) or that retromer has a different mechanism of action with non–BAR-containing adaptors. We set out to resolve this contradiction by determining the architectures of metazoan and fungal retromer coats with non-BAR adaptor proteins.

## RESULTS AND DISCUSSION

### The structure of the retromer:SNX3 membrane coat

We expressed and purified recombinant metazoan and fungal retromer core trimer, as well as the membrane adaptors, metazoan SNX3, and its fungal homolog Grd19. SNX3/Grd19 contains no BAR domain, consisting only of a phox homology (PX) domain that selectively binds phosphatidylinositol 3-phosphate [PI(3)P] (*22*). Each core trimer was reconstituted with its respective adaptor on membranes containing the early endosomal marker phospholipid PI(3)P (*23*) and cargo peptides (fig. S1A) derived from the C-terminal domains of Wntless (Wls) for metazoa (*11*, *24*, *25*) or the Ca^2+^-dependent serine protease Kex2 (*26*–*28*) for fungi. Cryo–electron microscopy (EM) imaging of these membrane-reconstituted complexes revealed the formation of abundant long tubules with a dense protein coat (fig. S1B) immediately demonstrating that SNX-BARs are not required for tubulation of the underlying membrane.

**Fig. 1:**
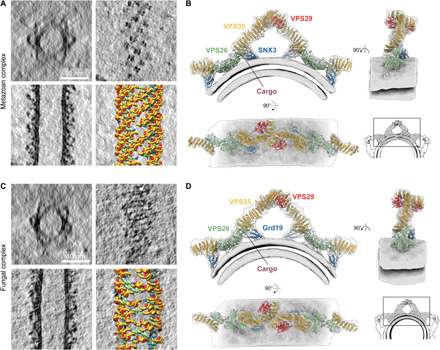
Overview of metazoan and fungal retromer:SNX3 coats assembled on membranes in the presence of cargo peptides. (**A**) Representative slices through tomographic reconstructions of metazoan retromer:SNX3-coated membrane tubules. Top left: radial cross section through a tubule. Top right: axial cross section at the level of arch heads. Bottom left: axial cross section through the middle of a tubule. Bottom right: the slice from the bottom left overlaid with models of coat subunits placed in space according to their positions and orientations found through subtomogram averaging. Models colored as in (B). (**B**) Overlay of ribbon-depicted atomic models color-coded by protein, and composite EM map covering the membrane, arch, and two neighboring VPS26 dimer regions with short segments of VPS35 of the next arches. The grayscale inset shows a model of a section of a tube prepared by overlay of three composite maps. (**C** and **D**) As in (A) and (B) for the fungal retromer:Grd19 complex.

We imaged the protein-coated tubules by cryo–electron tomography and applied reference-free subtomogram averaging to generate EM density maps of the assembled coat. Initial alignments showed the presence of arch-like units similar to those previously observed in fungal retromer:SNX-BAR coats and on protein-coated tubules within green algae (*6*). Using local alignment, we produced overlapping maps (fig. S2) that revealed details of the fully assembled cargo-containing coats of metazoan retromer:SNX3 and fungal retromer:Grd19 at subnanometer resolution (Fig. 1). Consistent with the measured resolution (fig. S2), protein secondary structure elements were readily resolved in the EM maps, allowing unambiguous docking of experimental and homology atomic models. Comparison of the assemblies demonstrated that metazoan (Fig. 1B) and fungal (Fig. 1D) coats are essentially identical, and hereafter, we describe metazoan retromer:SNX3 unless stated otherwise. The coat consists of arch-like units formed by VPS35 homodimerization that are directly connected to membrane-attached assemblies of VPS26 dimers and two SNX3 molecules (Fig. 1, B and D) contradicting recent assertions that the VPS35 arch is incompatible with SNX3 membrane binding and that retromer:SNX3 must therefore adopt a flat structure (*21*).

Arches were arranged in a pseudohelical lattice wound around membrane tubules (Fig. 1, A and C). The fungal retromer:Grd19 lattice shows less regularity than either the metazoan retromer:SNX3 or the previously described fungal retromer:SNX-BAR lattice (*6*). Differences in regularity could result from differences in the adaptor (for example, because of SNX-BARs forming regular lattices themselves), from differences in cargo (for example, Wls contains two cargo-binding motifs that could cross-link retromer trimers, while Kex2 contains only one), or from subtle differences in the protein-protein interactions that influence the dynamics of the assembly process.

### Membrane interactions of the retromer:SNX3 coat

**Fig. 2:**
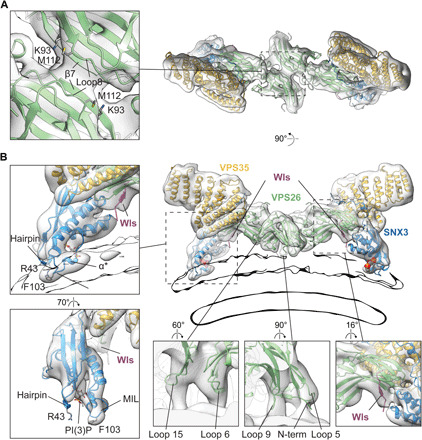
Structure of the membrane-proximal region of the metazoan retromer:SNX3 complex. Ribbon models colored by subunit and fitted within semitransparent EM density maps. Modeled PI(3)P is shown as spheres (in overviews) or stick model (in close-up views). (**A**) View looking “down” onto the membrane. Inset shows a close-up of the VPS26 homodimerization interface, marking secondary structure elements forming the interface and residues with PD-associated mutants. (**B**) View perpendicular to membrane. Insets show regions indicated by boxes. The membrane is shown as an outline of the same EM map at lower contour level. Two left-hand panels illustrate features involved in SNX3 membrane anchoring including the membrane-submerged MIL, α*, and bound PI(3)P. The conserved R43 and F103 within the MIL are marked. Bottom panels demonstrate membrane-contacting VPS26 loops (left and middle panels shown at lower contour level than the overview) and EM density for the Wls-occupied cargo-binding site (right). Equivalent views for the fungal retromer:Grd19 complex are shown in fig. S3B.

In the assembled retromer:SNX-BAR, the core trimer does not contact the membrane but is bound to the SNX-BAR adaptor layer via loops 5 and 9 of VPS26 (*6*). In contrast, the core trimer in retromer:SNX3 coats docks directly to the membrane, and we now see the structural details of this docking (Fig. 2): Loops 5, 6, 9, and 15 and the N terminus of VPS26 directly contact the membrane (Fig. 2B and fig. S3A). Phosphorylation in VPS26 loop 6 was previously shown to influence the trafficking of chitin synthase 3 in yeast (*29*). Structurally, VPS26 loops 6 and 15 are equivalent to the β-arrestin “finger” and “C-loop,” which interact directly with the transmembrane core and membrane proximal residues of cargo receptor (*30*). In the context of retromer:SNX3 coat, loops 6 and 15 of VPS26 have access to the membrane, and we speculate that they play a similar role interacting with transmembrane domains of cargoes as described for TGN38 in metazoa (*31*) and Snc1 in yeast (*32*).

To engage with sorting motifs located on the unstructured cytoplasmic tail of cargo, VPS26 adopts an “open” form where the outward movement of the β10 strand generates the interface for cargo binding (*33*) as opposed to the closed conformation observed in the absence of cargo (fig. S4) (*34*). Previously, this interface was described by crystallizing a minimal part of the retromer complex with a cargo motif covalently linked to the VPS26 as a chimera (*33*). For both metazoan and fungal retromer:SNX3 coat reconstitutions, we included membrane-attached cargo peptides that contain a canonical *Øx*(*L*/*M*) sorting motif (where *Ø* is an aromatic amino acid and *x* is any residue). In both structures, we observed that VPS26 is in the open conformation (fig. S4) and that density corresponding to the cargo peptide (Wls and Kex2, respectively) is visible on VPS26 near the SNX3-binding interface (Fig. 2B and fig. S3B), indicating that this site can bind cytoplasmic portions of cargoes in a physiologically relevant, membrane-assembled state.

VPS26 forms a homodimer on the membrane by β sheet complementation between the β7 strands of the N subdomains, creating an interface identical to that seen in the retromer:SNX-BAR coat (*6*). VPS26 homodimerization is therefore not specific to interaction with a SNX-BAR layer as has been suggested (*21*). Loop 8, which is positioned above β7, is also likely involved in the stabilization of VPS26 dimers (Fig. 2A). Several mutations in VPS26 (K93E and M112V/M112I) occur in sporadic and atypical Parkinson’s disease (PD) with an unknown causative mechanism (*35*). The coat assemblies described here place these mutations in close proximity to the VPS26 homodimerization interface (Fig. 2A) where they will likely impair VPS26 dimerization and hence coat formation providing an explanation for the mutations’ pathophysiological effects.

In the structure presented here, SNX3 binds the core trimer at the interface between VPS26 and VPS35. It is oriented with the PI(3)P-binding pocket facing the membrane and with a clear density for the PI(3)P head group present in the pocket (Fig. 2B). SNX3 is further anchored in the membrane via the membrane insertion loop (MIL) containing the short α helix α* (*36*), and the β1-β2 hairpin, which contains a membrane-facing arginine (Fig. 2B and fig. S3A). The fungal Grd19 is similarly anchored, although it has a shorter MIL compared to SNX3 and lacks α* (fig. S3, B and C). A sequence alignment of PX domains from all known SNX family adaptors confirms that, although variable in length, the MIL is a consistent feature of membrane-interacting PX domains (fig. S3, C and D). In contrast, in PX domains of SNX5 and SNX6 that are unable to bind membranes (*22*), the MIL is replaced by an extended helix-turn-helix structure involved in cargo binding (fig. S3, C and D). To perform their function in cargo binding and sorting, SNX5 and SNX6 form heterodimers with either SNX1 or SNX2 (*37*), both of which have MIL-containing PX domains.

### The retromer arch is asymmetric and conserved

**Fig. 3:**
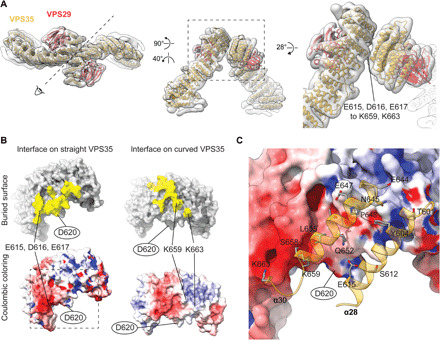
Conserved, asymmetric VPS35 dimerization interface. (**A**) Top and side views of ribbon representation of flexibly fitted VPS35/VPS29 color-coded models into the EM map of the metazoan arch. The direction of view presented in the middle panel is indicated in the left panel. The right-hand panel shows a close-up of the area boxed in the middle panel. The EM density corresponding to the electrostatic interaction between E615, D616, and E617, and K659 and K663 is indicated. (**B**) Cutaway views of the dimerization surfaces of straighter and the more curved VPS35 molecules. Model-generated surfaces are colored by the buried interface in yellow (top) or by coulombic potential from red (negative) to blue (positive) (bottom). Key electrostatic residues and D620, which is mutated in PD (oval outline), are indicated. To produce the interface model preserving experimentally determined side-chain orientations, the atomic model of the C-terminal region of human VPS35 [PDB 2R17:B (*70*)] was docked as a rigid body into the EM densities of the helices facing the interface of both VPS35 molecules. (**C**) Close-up view of the surface model of the interface on the straight VPS35 [boxed area in (B)] overlaid with ribbon model of key contact helices α28 and α30 in the curved VPS35 with a stick representation of residues within the buried interface.

Using local reconstruction and three-dimensional (3D) classification, we found that, for both metazoan and fungal retromer:SNX3 datasets, the arch is asymmetrical (Fig. 3). One VPS35 monomer is more curved than the other, and the two monomers interact via an asymmetric VPS35:VPS35 dimerization interface. This asymmetry causes the arches to tilt by approximately 22° away from the perpendicular relative to the membrane (Fig. 1, B and D). We reprocessed the retromer:SNX-BAR dataset (fig. S2A) where we had previously applied twofold symmetry and found that it, in fact, also uses the same asymmetric VPS35 dimerization interface (fig. S5). The interface is formed predominantly by the loops between α28-α29, α30-α31, and α33-α34 from the straighter conformation of VPS35 binding to α30 and α28 of the more curved VPS35 molecule (Fig. 3 and fig. S5). The interface relies on electrostatic and hydrophobic contacts between highly conserved residues with a prominent electrostatic bridge formed between E615, D616, and E617 on straighter VPS35 and K659 and K663 on the more curved VPS35 (Fig. 3, B and C; and fig. S6, C and E). The combination of those residues was confirmed biochemically to be crucial for the formation of dimerization interfaces (*21*); however, their proposed model of dimerization was different (fig. S6, D and E). VPS35L, which is predicted to be the VPS35 homolog in the retriever complex (*1*, *38*), does not contain the critical dimerization residues (fig. S6E), suggesting that VPS35L is either unable to homodimerize or that the interactions at the dimerization interface are different.

Asymmetric VPS35 dimerization has possible implications for retromer function. It leads to an asymmetric VPS35 surface at the apex of the arch to which cofactors could bind directionally and with a stoichiometry of 1 cofactor:2 VPS35 molecules. For example, a D620N mutation that is associated with familial autosomal dominant and sporadic PD (*39*) causes loss of affinity to the family with sequence similarity 21 (FAM21) protein (*40*), the retromer-binding subunit of Wiskott-Aldrich syndrome protein and SCAR homolog complex (WASH) (*41*). D620 is part of the buried interface in straighter VPS35, where mutation may disrupt the coat. D620 is, however, exposed in the more curved VPS35 (Fig. 3, B and C), where it may contribute to a single FAM21-binding site at the apex. The interfaces of the two VPS29 subunits at the apex of the arch may also be differently accessible for binding by regulator factors such as the Tre-2/Bub2/Cdc16 domain family member 5 (TBC1d5) (*42*), Vps9-ankyrin-repeat protein (VARP) (*43*), and RidL, the effector of pathogenic bacteria *Legionella pneumophila* (*44*).

Asymmetry could also impose directionality of coat assembly. This could be a direct effect if the interface on one side of the arch provides a better substrate for further coat oligomerization than the different interface on the other side. It could also be an indirect effect, if a cofactor that binds asymmetrically to the asymmetric arch exposes an interface in one direction that promotes oligomerization. We note that coat protein complex II (COPII), which is thought to assemble tubular carriers for the transport of large cargoes (*45*, *46*), can form tubular arrays in which polarity is defined by a membrane-bound Sec23/24/Sar1 array (*47*, *48*).

### Retromer consists of a conserved scaffold and alternative adaptor modules

**Fig. 4:**
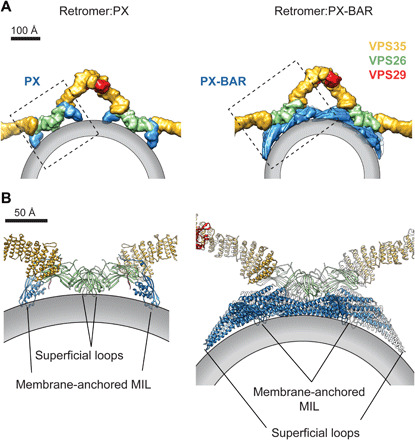
Modularity of the retromer coat allows distinct modes of membrane attachment using different adaptors. (**A**) Surface model illustration of retromer:PX (modeled on metazoan retromer:SNX3) and retromer:PX-BAR (modeled on fungal retromer:Vps5). Surface models were generated from pseudoatomic models of corresponding complexes and color-coded. The membrane bilayer is schematically shown as a gray outline. (**B**) Close-ups of the areas boxed in (A) showing the assembly of membrane-attached subunits in each complex. The VPS26/SNX3 assembly (left) and the four Vps5 dimers contacted by the VPS26 dimer (right) [PDB 6W7H (*6*)] have inherently curved structures that use a combination of membrane-anchored MILs (from the PX domains of SNX3 and Vps5) and superficially attached loops (from VPS26 and the tip loops of the BAR domain) to contact the membrane.

The recent observation that retromer forms lower-order oligomers on bilayers with limited flexibility (*49*) suggests that higher-order oligomerization and membrane remodeling are interconnected processes. We observed that, on flexible membranes, retromer forms tall arches and not the recently suggested flat structures (fig. S6, A and B). Arches are assembled into linear chains via two homodimerization interfaces—the asymmetrical VPS35 dimer interface and the symmetrical VPS26 dimer interface (Fig. 1). This mechanism of coat formation is invariant in the retromer coats that we have studied, independent of kingdom (animals, fungi, and plants), and independent of adaptor (with or without BAR domain) (Fig. 4 and fig. S7). This invariable arched scaffold can use two distinct membrane coupling modes to accommodate different adaptor types: It can bind either directly to the membrane (retromer:PX) or via the BAR layer (retromer:PX-BAR) (Fig. 4A). The direct membrane-binding mode can be observed for retromer arches within cells (fig. S7). We can now directly compare coats with different membrane-binding modes to understand the mechanism of the membrane tubulation.

The structures suggest how retromer may achieve membrane tubulation by combining multiple mechanisms. The core trimer arch—the conserved architectural element of the different retromer coats—forms a scaffold that can contribute directly or indirectly to membrane bending and which propagates curvature over larger areas of membrane by oligomerization. In retromer:PX-BAR coats, membrane bending will be enhanced through the well-established local membrane-remodeling action of the curved BAR domains (Fig. 4B). In the case of retromer:PX coats, membrane bending will be enhanced through a similar local membrane remodeling by the curved subcomplex of a VPS26 homodimer and two SNX3 proteins (Fig. 4B), while further membrane remodeling derives from insertion of loops from VPS26 and SNX3 into the head group layer of the membrane. This bears similarities to the COPI and COPII coats, which also remodel membranes through a combination of a curved scaffold and membrane insertion (*47*, *50*).

This model also permits retromer assemblies with other non–SNX-BAR adaptors such as SNX12 and SNX27 that may have similar membrane-remodeling properties to SNX3 and is consistent with the previous observation that retromer promotes the membrane-remodeling activity of yeast Grd19 (*51*). It suggests a general modular scheme for retromer function in which the core trimer can make use of a range of different adaptor modules that determine the cargo to be incorporated and in which both the core trimer and the adaptor contribute to membrane curvature. In this way, the adaptor can modulate the degree of membrane bending appropriate to the size of the cargo it incorporates and/or the bilayer properties along the trafficking route.

## MATERIALS AND METHODS

### Protein expression and purification

All proteins were expressed at 20°C in *Escherichia coli* BL21(DE3) following induction with 0.2 mM isopropyl-β-d-1-thiogalactopyranoside as previously described in (*6*). Glutathione *S*-transferase (GST)–tagged proteins were constructed in pGEX-4T2 by genetically fusing fungal (*Chaetomium thermophilum*, Ct) CtGrd19 (UniProt G0S0X3), mouse SNX3 (UniProt O70492), and zebrafish VPS26 (UniProt Q6TNP8) to the C terminus of GST using a polymerase chain reaction–based cloning method (*52*). It allowed us to introduce an additional cleavage site for the PreScission protease located downstream of the thrombin site. Fungal core trimer was purified as described in (*6*). The mouse GST-VPS35 (UniProt Q9EQH3):VPS29 (UniProt Q9QZ88) heterodimer was expressed and purified as in (*53*). All GST-containing proteins that we purified followed the standard protocol (*54*). In short, cells were lysed by high-pressure homogenization in 50 mM tris-HCl (pH 8.0) and 200 mM NaCl buffer. The homogenate was cleared by centrifugation at 30,000*g* and loaded onto a gravity flow column containing Glutathione Sepharose 4B (GE Healthcare). The proteins were eluted by protease cleavage of the GST tag [thrombin or PreScission (Sigma-Aldrich) were used, depending on the enzyme availability] and were further purified by gel filtration chromatography on Superdex 200 10/300 column (GE Healthcare) in buffer A [20 mM Hepes-KOH (pH 7.5), 200 mM NaCl, and 1 mM tris (2-carboxyethyl)phosphine].

His-tagged cargoes were constructed in pRSFDuet-1 by genetically fusing residues 733 to 846 of Kex2 (UniProt G0SHU5) and residues 493 to 541 of Wls (UniProt Q6DID7) to the fusion tag, resulting in His_10_Kex2 and His_6_Wls, respectively. The cargo tail peptides were isolated on a gravity flow column containing Ni–nitrilotriacetic acid agarose (Ni-NTA) (GE Healthcare) followed by gel filtration chromatography on Superdex Peptide 10/300 (GE Healthcare) in buffer A.

### Liposomes and tubulation reactions

Liposomes composed of 1,2-dioleoyl-sn-glycero-3-phosphocholine (DOPC), 1,2-dioleoyl-sn-glycero-3-phosphoethanolamine (DOPE), 1,2-dioleoyl-sn-glycero-3-phospho-l-serine (DOPS), and 1,2-dioleoyl-sn-glycero-3-[(*N*-(5-amino-1carboxypentyl)iminodiacetic acid)succinyl] (DGS-Ni-NTA) nickel salt (all Avanti Polar Lipids) in a 42:42:10:3 molar ratio, with 3 mole percent of dipalmitoyl-phosphatidylinositol-3-phosphate [PI(3)P] (Echelon Biosciences), were prepared at a lipid concentration of 0.5 mg/ml in buffer A by extrusion through a 0.4-μm polycarbonate filter (Avanti Polar Lipids). For the tubulation reaction, 2.5 μM of the core retromer trimer, 3.5× molar excess of the adaptor (SNX3 or Grd19), and the corresponding cargo peptide (Wls or Kex2) were incubated with liposomes (0.16 mg/ml) for 4 hours at 22°C in buffer A.

### Cryo–electron tomography sample preparation and data acquisition

Gold fiducial markers (10 or 5 nm) (BBI Solutions) in buffer A were added to the tubulation reaction (1:10 fiducials:reaction volume ratio). Four microliters of this mixture was backside-blotted for 6 s at a relative humidity of 98% and a temperature of 19°C on a glow-discharged holey carbon grid (CF-2/1-3C; Protochips) before plunge-freezing in liquid ethane (Leica EM GP2 automatic plunger). Dose-symmetrical tilt series acquisition (*55*) was performed on an FEI Titan Krios electron microscope operated at 300 kV using a Gatan Quantum energy filter with a slit width of 20 eV and a K2 or K3 direct detector operated in counting mode. The total exposure of ~130 e^−^/Å^2^ was equally distributed between 41 tilts. Ten frame movies were acquired for each tilt. The details of data collection are given in table S1. The selection of acquisition areas was guided by suitability for high-resolution tomographic data collection (i.e., vitreous ice quality, lack of crystalline ice contaminations, and intactness of the carbon support) and was not based on the morphology of tubules.

### Tomogram reconstruction

Image preprocessing and tomogram reconstruction were performed essentially as described in (*56*). The IMOD v. 4.10.3 package (*57*) was used to align frames in raw movies and correct for detector gain and pixel defects. Several tilt series in each dataset were discarded at this point (dataset sizes and all exclusions are listed in table S1) due to tracking errors or large beam-induced sample movements. In addition, defective high-tilt images (due to tracking error, large objects like a grid bar, or contaminations coming in the field of view) were also removed before low-pass filtering to the cumulative dose (*58*). Tilt series were aligned on the basis of the fiducial markers in the IMOD package. The aligned tilt series were binned four times and reconstructed by weighted back projection in IMOD, resulting in the tomograms uncorrected for contrast transfer function (CTF) that were used for visual inspection of the quality of tomographic reconstruction, tubule picking, and defocus estimation using CTFPLOTTER (within the IMOD). To reconstruct 3D CTF-corrected tomograms for subtomogram averaging, dose-filtered tilt series were CTF-corrected by phase-flipping and back-projected into tomographic reconstructions using NovaCTF (*59*) with a 15-nm strip width. The resulting unbinned tomograms were binned by factors of 2 (generating bin2, bin4, and bin8 tomograms) with antialiasing using IMOD.

### Subtomogram alignment

Subtomogram alignment and averaging were done as previously described in (*6*, *60*) using MATLAB (MathWorks) functions adapted from the TOM (*61*) and AV3 (*62*). The scripts and relevant documentation (“SubTOM” package, v1.1.4) are available to download via the following links: www2.mrc-lmb.cam.ac.uk/groups/briggs/resources and https://github.com/DustinMorado/subTOM/releases/tag/v1.1.4. As in (*56*), we used a modified wedge mask representing the amplitudes of the determined CTF and applied exposure filters at each tilt (*60*, *63*). Table S2 contains a summary of data processing parameters. Subtomogram averaging was performed identically and independently for the two datasets as described in the following sections.

### Extraction of initial subtomograms

Centers of coated tubules were manually traced in bin4 tomograms, and their diameters were recorded using Chimera (v1.14) and the custom plug-in (*64*). All tubules were included (no selection was made for diameter or morphology) (table S1). The positions of subtomograms were defined on the surface of tubes with uniform sampling at every 44 Å. Initial subtomogram orientations were set to be normal to the membrane surface and with the in-plane angle perpendicular to the main tubule axis.

### Ab initio reference generation

Subtomograms were extracted at the initial positions from bin8 tomograms and averaged according to their initial orientations, producing an initial reference. Subtomograms were then aligned to this reference in the direction perpendicular to the membrane and averaged to generate a reference containing density layers corresponding to the lipid bilayer and protein layer. An arbitrary subset of the data (10 tomograms) was then further iteratively aligned, allowing both shifts and angular search. A cylindrical mask was applied passing the protein layer and the membrane. Five iterations of such alignment were performed with an 8° to 4° angular search increment and a 40-Å low-pass filter. The resulting average was shifted and rotated to place the arch or the VPS26 dimer in the center of the box.

### Subtomogram alignment and classification

The resulting references were used to align the complete dataset with similar alignment parameters. Overlapping subtomograms resulting from oversampling at the initial extraction stage were removed by selecting the subtomogram with the highest cross-correlation score within a distance threshold of 55 Å. Subtomograms were then split by tubule into odd and even half datasets for further processing. Subsequent alignments were performed independently on the odd and even half-sets. The search space and angular increments were gradually decreased, and the low-pass filter was gradually moved toward higher resolution. After visual examination, misaligned subvolumes (those not aligned to the membrane) were removed using a cross-correlation cutoff that was manually selected for the whole dataset. In some cases, subvolumes located close to the edge of tomograms were also removed. At the end of each iteration, subtomograms within each half-set were averaged, and resolution was assessed by Fourier shell correlation.

3D classification was performed using principal components analysis of wedge-masked difference maps (*65*) with calculations implemented in the SubTOM package (v1.1.4) using code adapted from TOM (*61*) and AV3 (*66*). An identical workflow was used for reprocessing of retromer:Vps5.

### EM map postprocessing

Final half-maps were filtered with soft masks to remove the box edge using IMOD (4.10.3) and EMAN (2.2.2) packages. Local resolution was measured using relion_postprocess from the Relion 3.0.8 package (*67*). The final sharpened maps were prepared using local resolution filtering and denoising implemented in LAFTER (v1.1) (*68*).

### Model building

Rigid-body fitting was performed in Chimera, peptide linkers were built using Modeller (version 9.24), and flexible fitting used the Real-Space Refine procedure in Phenix (1.18.2-3874). SWISS-MODEL (*69*) web service was used for homology modeling. Chimera and ChimeraX (v1.0) packages were used for molecular visualization.

To build models of the arch, structures of human VPS29 [Protein Data Bank (PDB) 2R17:A] and N- and C-terminal portions of VPS35 (PDB 5F0L:A and 2R17:B, respectively) were fitted into the EM map of the metazoan arch region as rigid bodies. The missing linker between the two portions of VPS35 was built, and the resulting full-length VPS35 model was flexibly fit with secondary structure constraints to account for movements of individual helices occurring along the length of α-solenoid. The fungal model was built using an identical procedure, but based on a homology model of CtVPS35 (UniProt G0S709) generated from the above human VPS35 models together with the experimental model of CtVps29 (PDB 5W8M).

To build models of the VPS26 dimer region, two copies of the experimental model of human VPS26/SNX3/N-termVPS35/DMT-II cargo peptide assembly (PDB 5F0L) were rigidly fitted into the metazoan EM map. They were then split into VPS26/DMT-II, SNX3, and N-terminal VPS35 subunits, and the N-terminal VPS35 subunit was flexibly fitted into the arch EM map as described above. Subunit positions were refined by rigid-body fitting using sequential fit command in Chimera. The PI(3)P model was copied into SNX3 from the yeast homolog Grd19p (PDB 1OCU) complexed with PI(3)P. Modeling into the fungal EM map was done identically, but the human subunits (excluding the DMT-II cargo peptide) were replaced with the corresponding *Ct* homology models. CtGrd19 and CtVps26 were modeled on yeast 1OCU and human 5F0L PDB models, respectively.

## SUPPLEMENTARY MATERIALS

Supplementary material for this article is available at http://advances.sciencemag.org/cgi/content/full/7/13/eabf8598/DC1

## Appendix

View/request a protocol for this paper from *Bio-protocol*.
